# Contributions to Midwifery and Diseases of Women and Children; with a Report on the Progress of Obstetrics, and Uterine and Infantile Pathology in 1858

**Published:** 1859-11

**Authors:** 


ART. IV.—*Contributions to Midwifery and Diseases of Women and Children; with a Report on the Progress of Obstetrics, and Uterine and Infantile Pathology in* 1858. By E. NOEGGERATH, M. D., and A. JACOBI, M. D., New York: Ballière Brothers, 440 Broadway. 466 pp.

The original contributions by the authors are seven articles, occupying 105 pages of the volume. Our limits will not admit of a special notice of each; and we therefore shall simply remark, that the essay on the Etiological and Prognostic Importance of the Premature Closure of the Fontanelles and Sutures of the Infantile Cranium, is especially valuable and interesting, and is worthy of a most careful perusal. It is sufficient to stamp its author as a most industrious and philosophical observer, who is conscientiously working for the advancement of science. The remainder of the volume is filled with a Report on Obstetrics and Uterine Pathology, and a Report on Infantile Pathology. These reports are made up by abstracts from all the Manuals and Reports on these subjects which have appeared during the year 1858, and by a digest of the articles which have appeared in one hundred and nine medical journals. This list of course includes a great majority of the American, British, and Continental journals. The abstracts seem to have been made with judgment and impartiality, and great comprehensiveness, covering almost the whole domain of Obstetrics and Uterine and Infantile Pathology. The authors have occasionally added brief comments of their own, characterized by good sense, clothed sometimes in an amusing, but not offensive, Teutonic idiom. We like the plan of the book, and hope that the authors may be encouraged to continue their labors and make their Report an Annual. It should be in the hands of all who mean to keep up with the progress of science in these departments. Indeed, we may say that it is indispensable for such; for few have the time, the ability, or the opportunity to consult all the original sources from which the book is made up.

